# Relational Framework of Cyberattacks: Empirical Evidence from Multistage Incidents †

**DOI:** 10.3390/s25237124

**Published:** 2025-11-21

**Authors:** Mikel Ferrer-Oliva, José-Amelio Medina-Merodio, José-Javier Martínez-Herraiz, Carlos Cilleruelo-Rodríguez

**Affiliations:** Departamento de Ciencias de la Computación, Universidad de Alcalá, 28871 Madrid, Spain; mikel.ferrer@uah.es (M.F.-O.); josej.martinez@uah.es (J.-J.M.-H.); carlos.cilleruelo@uah.es (C.C.-R.)

**Keywords:** cyberattack taxonomy, threat progression, intelligence sharing, relational model, dark web

## Abstract

The increasing scale and operational complexity of cyberattacks have exposed the limitations of static taxonomies for representing multistage threat scenarios. This study addresses the need for more flexible classification models by proposing a relational taxonomy of cyberattacks grounded in documented incidents. Therefore, the main objective is to propose a relational taxonomy that encodes direct transitions across eight groups in a dependency matrix and a directed graph while preserving traceability to MITRE ATT&CK. The taxonomy was validated by an independent panel of experts who assessed methodological clarity and operational utility. The results reveal consistent transition patterns across groups, delineate reproducible escalation routes, and pinpoint cut-off points linked to specific detection and control activities, providing an operational map of progression and intervention. The conclusions show that the taxonomy clarifies escalation paths, strengthens alignment across security monitoring and incident response functions, threat intelligence workflows and training, and provides an operational structure to manage interdependencies, anticipate escalation and focus monitoring on critical points.

## 1. Introduction

The growing complexity of cyber threats has driven the development of taxonomies to classify and understand cyberattacks, which have become reference tools to structure knowledge, support risk analysis, and inform context-appropriate defensive strategies [1,2,3]. Consistent with this role, taxonomies enable communication and coordination by providing a shared language, a common structure, and cross-team traceability, in alignment with practitioner-oriented frameworks [4,5]. In vertical domains such as the IoT (Internet of Things), specialised surveys highlight both the scale of the phenomenon and the detection and classification challenges, reinforcing the value of rigorous taxonomic approaches [6].

The literature also identifies limitations of largely static taxonomies in scenarios where attacks do not follow linear patterns or predefined phases, thereby constraining their ability to represent dynamic sequences and functional relationships between techniques [7,8]. These constraints hinder the anticipation of tactical chains and the explanation of immediate operational dependencies between contiguous events in the offensive cycle [9,10].

This challenge is more acute in opaque ecosystems such as the dark web, where actors combine techniques in modular and adaptive ways and where the indicators available to defenders often lack the relational context required for timely decision-making; empirical studies and public datasets document this modularity and its relevance for collaborative cyber threat intelligence [11,12]. In parallel, methodological research underscores the need for requirements and criteria that make taxonomies more interoperable, actionable, and relationship-sensitive [13], for explicit evaluation of CTI (cyber threat intelligence) actionability across different consumer types [14].

Therefore, the main objective of this study is to propose a multistage relational taxonomy that represents immediate transitions between attack-technique groups and supports the analysis of operational dependencies, thereby improving the interpretation of complex incidents while remaining compatible with widely adopted frameworks such as MITRE ATT&CK [15].

The remainder of the manuscript is structured as follows. Section 2 presents the literature review and theoretical framework. Section 3 introduces the relational taxonomy and details the eight attack groups, the dependency matrix, and the graphical framework. Section 4 provides evidence by mapping real cases to one-step transitions. Section 5 describes the application methodology, including the acceptance checklist and alignment with MITRE ATT&CK. Section 6 reports the results of the taxonomy’s validation by the panel of experts. Section 7 presents the discussion of the results obtained. Section 8 shows the main conclusions obtained.

## 2. Literature Review and Theoretical Framework

The analysis and classification of digital threats requires a structured approach that enables understanding not only the individual nature of each technique but also its evolution, interdependencies, and operational context. This section develops a theoretical framework composed of three interrelated themes that follow a coherent narrative, progressing from foundational concepts to the most recent proposals.

It begins with a review of the main traditional taxonomies and classification schemes used in cybersecurity, which have served to structure knowledge on threats over recent decades. Building on this foundation, the inherent limitations of static models are introduced, and the need to move towards more adaptive relational approaches is argued. Finally, dynamic proposals are addressed that aim to represent the tactical progression of attacks in real-world scenarios, including those specific to opaque environments such as the dark web.

### 2.1. Cybersecurity Taxonomies and Traditional Threat Classification

Threat classification in cybersecurity has been an essential tool for structuring the technical and operational knowledge associated with digital risks [4]. Over the past two decades, numerous taxonomies have been proposed to organise incidents according to criteria such as initial vectors, attacker objectives, types of techniques employed, or the impact generated [6]. This systematisation has improved communication among security teams, facilitated risk analysis, and laid the groundwork for coordinated incident response in complex environments [3].

One of the most influential reference frameworks has been MITRE ATT&CK [15], which catalogues tactics and techniques used in adversarial campaigns, structured according to the phases an attacker may follow after initial access. Its adoption has become widespread across both public and private sectors, consolidating its position as a de facto standard in many cyber intelligence operations [5]. However, limitations have been identified regarding its applicability to non-linear, scalable attack chains or those combining techniques from different vectors [7].

In this context, recent proposals have introduced automated methods to enrich these static taxonomies with dynamic operational data [16,17]. For instance, an approach has been developed to map CVE vulnerabilities directly to ATT&CK tactics using machine learning models, enabling automated identification of security gaps and reducing reliance on manual intervention in the analysis process [8].

The operational value of a taxonomy also depends on its ability to structure threat intelligence. Tools such as MISP [18] have promoted collaborative sharing of indicators of compromise (IoCs), and have been enhanced by proposals like CARIOCA, which prioritises indicators based on their relevance and context, thereby improving the traceability and effectiveness of alerts [19]. Additionally, standards such as STIX 2.1 [20] and TAXII [21] have been pivotal in standardising the representation and distribution of cyber threat intelligence, allowing for the structuring of complex relationships between threats, techniques and targets, and facilitating automated sharing among organisations.

The degree of “actionability” of such intelligence has also been subject to review. Proposals such as EVACTI [14] have introduced evaluation models that estimate the practical value of a STIX [20] report for different consumers, using objective criteria inspired by European cyber intelligence frameworks. This allows for assessing the real utility of a piece of information beyond its syntactic formalisation, extending the functional value of traditional taxonomies [14].

Despite these advances, structural barriers persist in classification systems. One of the most cited issues is the coexistence of multiple taxonomies with different structures, terminologies and approaches, which hampers interoperability and leads to redundancy or overlap [22]. This fragmentation has been documented in comparative studies that have mapped taxonomies oriented towards risks (VERIS), behaviour (Kill Chain) or adversarial relationships (Diamond Model), emphasising the need for unified criteria and shared properties to enable convergence [3,23,24,25].

These limitations also affect the extrapolation capacity of existing models [26]. Most current taxonomies have been developed within information technology contexts, which restricts their application to more demanding domains such as cyber–physical systems (CPSs). In this area, adaptive approaches have been proposed to represent threats such as ransomware in CPS, incorporating technical vectors, industrial targets and specific tactics observed in real incidents, such as the Colonial Pipeline case [27].

The technical literature shows a growing consensus on the need to move towards more dynamic, interconnected and relationship-based taxonomies [28,29,30]. While static approaches are useful for mapping individual stages of an attack, they prove insufficient when it is necessary to represent the progression of a threat, its interaction with other techniques or its functional chaining within persistent campaigns [31]. The integration of relational elements, contextual prioritisation and causal links thus emerges as a key requirement for improving systemic understanding of digital risk [8,14,27].

### 2.2. Limitations of Static Taxonomies and the Need for Relational Models

Cyberattacks rarely manifest as isolated actions; rather, they unfold as sequences in which each stage enables the next, forming trajectories that require relational and contextual analysis [9,10]. However, most current taxonomies are limited to classifying behaviours in a segmented manner, without representing the functional interdependencies between consecutive techniques [32]. This fragmentation constrains their utility in anticipating complex attack patterns or designing effective reactive defences [33].

In initial phases, threats typically exploit social engineering techniques such as phishing or vishing, which allow attackers to obtain credentials or introduce malware into target systems [34,35,36]. Subsequently, many campaigns combine multiple vectors, as seen in the case of the Lucifer malware, which integrates cryptojacking, DDoS attacks, and vulnerability exploitation within a single attack flow [37]. IoT botnets represent another case, where compromised devices are used to structure large-scale distributed attacks [38,39].

This progressive behaviour is difficult to represent using fixed taxonomic schemes. Most static frameworks, such as STIX 2.1 [20] and TAXII 2.1 [21], allow for the description of isolated indicators, but lack structures that link tactics based on causal dependency. Even collaborative platforms such as MISP [18], though useful for sharing intelligence, do not explicitly consider transitions between tactical phases or the enabling role that one technique may play in relation to another [40].

Recent research on taxonomy generation has highlighted the limitations of static models, which are often regarded as rigid, poorly adaptable and lacking operational scalability in dynamic scenarios [41]. There is also a documented need for structures capable of reflecting multiple dimensions, functional relationships between phases and practical applicability criteria, particularly in highly volatile contexts such as the dark web [13,41].

In the field of threat intelligence, significant shortcomings have been identified when relying exclusively on context-free indicators [42]. This isolated perspective has proven inadequate for integrating CTI into strategic decision-making, especially when speed and traceability are required in response to emerging threats [11,43]. In scenarios where information flows opaquely (such as clandestine markets or exploit forums), the absence of relational models may delay detection and undermine response quality [44].

Analysis of approaches applied to persistent campaigns has shown that traditional sequential models fail to adequately represent multistage attack sequences and adaptive variants [45]. In contrast, graph-based semantic structures have been proposed that can represent relationships among techniques, vulnerabilities and actors, allowing the construction of more accurate models for uncovering unidentified threats and managing complex dependencies [46].

In industrial environments, evidence suggests that hybrid models (which integrate system-centric, attacker-centric, and risk-centric perspectives) offer greater capacity to represent real-world scenarios and detect combined vulnerabilities [47,48]. This approach has proven particularly effective in identifying technical interdependencies that isolated models cannot capture [49]. Interorganisational coordination through platforms such as MISP has also proven effective in reducing response times, especially when addressing threats that emerge on the dark web or require collaborative intelligence for their characterisation [11].

### 2.3. Dynamic Taxonomies and Attack Progression in Real-World Scenarios

The tactical evolution of cyber threats requires classification models capable of reflecting complete attack chains and contextual relationships between events [50]. Static taxonomies (based on fixed hierarchical structures) are insufficient for capturing the operational dynamics observed in real-world environments, where techniques, targets and actors combine in non-linear ways [51,52]. This need becomes particularly acute in scenarios such as the dark web, where threats emerge in decentralised forms and with limited traceability, making them difficult to classify within conventional frameworks [53].

Multiple studies have demonstrated that forums, marketplaces and hidden platforms act as generators and amplifiers of cyber threats, promoting complex vectors such as ransomware, the sale of initial access, data leaks and crimeware-as-a-service campaigns [12,54]. The structured collection of information in these environments has led to the development of public datasets with strategic value, which reveal criminal patterns, recurring behaviours, and functional relationships between attack elements [55,56].

In response to these dynamics, taxonomies have been developed that combine technical and organisational sources through semantic graphs, enabling the representation of interactions among entities such as actors, tools, tactics, or compromised data [57]. These relational architectures make it possible to overcome the limitations of static schemes by capturing causal dependencies and changes in the threat over time. Some proposals even apply UML modelling or adaptive systems geared towards early detection and contextual classification [50,58].

AI-based solutions have also been proposed, including neural networks designed to monitor activities on the dark web and identify indicators of attack prior to their manifestation [59]. These tools analyse forums, marketplaces, and behavioural patterns to construct dynamic taxonomies focused on operational relationships [60]. This perspective has been successfully applied to the early detection of complex threats and the graphical representation of criminal sequences [61].

Recent studies have explored ransomware operations documented on the dark web, revealing interconnected structures of campaigns, cryptocurrency payments, extortion, and data leaks articulated as unified narratives [62,63]. Such evidence has highlighted the need for relational taxonomies capable of capturing the full progression of these attacks in real environments [64].

Additionally, some standardisation efforts (such as those promoted by the CASE model) have attempted to formalise cybersecurity events using structured ontologies [65]. However, the limited adoption of the standard and inactivity in its maintenance have constrained its practical application [66].

## 3. Proposed Cyberattack Taxonomy

Modern cyberattacks do not occur as isolated incidents but rather as chained sequences of techniques operating in conjunction [32]. This progression requires classification structures that can represent not only individual vectors but also the functional relationships between them [33]. The proposed model addresses this need through a classification structured into main groups, organised according to the objective of the techniques employed [16]. This segmentation enables the identification of escalation patterns, the anticipation of adversarial movements, and the design of more coherent defensive responses [34,67,68,69].

The following presents the eight defined attack groups, whose interactions will subsequently be represented in the form of a relational matrix (Section 3.2) and visually illustrated in the consolidated framework (Section 3.3).

### 3.1. Classification of Attack Groups

To structure the proposed taxonomy, eight cyberattack groups have been defined. These represent sets of techniques, vectors, and purposes sharing common operational patterns. This classification provides a coherent segmentation for relational threat analysis, particularly in real-world scenarios where attacks unfold sequentially or in combination [70].

- Identity and Authentication Attacks (IAAs): This group includes attacks focused on the acquisition and misuse of credentials, such as brute force, dictionary attacks, credential stuffing, or the reuse of leaked passwords [35,67].
- Social Engineering (SE): This includes psychological manipulation techniques aimed at inducing users to disclose information or perform unsafe actions. It constitutes one of the most frequent access vectors, with examples such as phishing, vishing, or baiting, whose effectiveness has been widely documented in both corporate and governmental contexts [34,36].
- Malware-Based Attacks (MBAs): This encompasses the use of malicious software to compromise the confidentiality, integrity or availability of systems. It includes everything from trojans and ransomware to hybrid botnets combining cryptomining with denial-of-service capabilities, as seen in the case of the Lucifer malware [37,71].
- Network Infrastructure Attacks (NIAs): Refers to attacks targeting critical network components such as servers, routers, and edge devices, with the aim of disrupting services, gaining lateral access or destabilising distributed infrastructures [31,38].
- Exploiting Software Vulnerabilities (ESVs): Encompasses actions that exploit flaws in software or operating systems to execute arbitrary code, escalate privileges, or access restricted resources. This group includes both known vulnerabilities and zero-day exploits [39,72].
- Attacks on Protocols and Communications (APCs): Focused on the interception or manipulation of communication channels using techniques such as Man-in-the-Middle, DNS hijacking or packet injection. These attacks compromise the integrity of data in transit [9,73].
- Advanced Persistent Threats and Cyberespionage (APT): Represents highly sophisticated and prolonged campaigns conducted by organised actors seeking stealthy and sustained access to sensitive assets, using advanced evasion and persistence techniques [24,68].
- Attacks on Critical IT/OT Infrastructure (CIIA): Refers to offensive actions targeting critical infrastructures such as industrial or maritime systems (including SCADA), where the impact may entail significant operational and economic consequences [69,74].

This segmentation captures both traditional attacks and emerging chained threats. It enables a clear depiction of typical intrusion flows persistent actor behaviours and combinations of techniques including supply chain compromises [75]. These types of attacks, due to their transversal nature, may manifest across multiple phases of the intrusion cycle, involving social engineering, malware, vulnerability exploitation and advanced persistence techniques [40,76]. Consistent with this technique level view, impacts that target databases are modelled within the existing functional groups rather than as a separate category.

### 3.2. Relational Dependency Matrix

In real-world operations the defined groups do not act in isolation. They operate in concert to sustain persistence and amplify the impact of the attack. This is especially true in environments such as the dark web, where attackers exchange tools and tactical knowledge, making such combinations more effective and complex [68,71].

Social engineering (SE) is a recurring entry point, enabling initial malware distribution and credential harvesting through techniques such as phishing or vishing [34,74,77]. For instance, spear phishing is commonly used to deliver modular malware that escalates privileges within compromised networks [35].

Malware-based attacks (MBAs) expand initial access using techniques such as ransomware, botnets, or cryptojacking, compromising additional systems and prolonging persistence within the network [37,38,72]. Certain ransomware variants have also incorporated capabilities for credential harvesting and lateral movement [71].

Exploiting software vulnerabilities (ESVs) involves leveraging flaws in outdated applications or systems to gain access or escalate privileges. Zero-day exploits have been particularly used in cyberespionage targeting government and industrial networks, allowing for prolonged undetected presence [70].

Communications and protocols (APCs) are also strategic targets for traffic manipulation and credential interception through techniques such as DNS hijacking or BGP hijacking, enabling the capture of sensitive data during transmission [9,39].

Compromising identity and authentication (IAA) facilitates both initial and secondary unauthorised access. Documented attacks have shown how credential stuffing enables malware installation without triggering internal security alerts, illustrating the effectiveness of compromised credentials as a stealth access mechanism [78]. The absence of multifactor authentication has also enabled unauthorised access to critical infrastructures [67].

Network infrastructure attacks (NIA) support lateral movement and deep infiltration, creating favourable conditions for persistent attacks and espionage or sabotage operations [31,40].

Advanced persistent threats (APT) frequently integrate multiple techniques, such as vulnerability exploitation and manipulation of industrial devices, to secure long-term presence, particularly in high-value government and corporate networks [24,68].

Finally, attacks on critical infrastructure (CIIA) have proven especially effective when combined with less sophisticated techniques, such as the use of stolen credentials. This allows dangerous interactions with operating and technological systems without needing to exploit complex vulnerabilities [79]. The exfiltration of sensitive data from such environments poses a direct threat to national and industrial security [68].

To represent these interconnections in real-world scenarios, a relational matrix has been designed in which each row indicates an initiating attack group and each column reflects the vectors that may be enabled from it. The initial attack is the tactic that establishes the enabling condition for the immediate next step within the same incident, and the facilitated attack is the immediate tactic whose execution becomes possible or significantly easier because of that condition. The matrix encodes a total of 20 relationships between the eight groups as one-step directed transitions from row to column. This structure enables the identification of frequent tactical flows and operational dependencies in real campaigns. It also shows how certain attacks act as entry vectors, for example, social engineering or credential compromise while others play a structural role in advanced phases such as persistent campaigns or attacks on critical infrastructures. The model is particularly useful in contexts such as the dark web where threats evolve in chained sequences and operational trajectories can be inferred from prior behaviour of malicious actors. Table 1 presents the relational matrix of direct dependencies among cyberattacks.

### 3.3. Taxonomy Framework

The graphical representation of the proposed model allows for visualising the tactical progression among the previously defined attack groups. This relational framework has been constructed based on the relationships encoded in the matrix from the previous section, adopting a directed graph structure. Each node represents an attack group, and each edge indicates a likely or documented transition between techniques, grounded in empirical evidence from real-world campaigns.

The graph reveals common patterns in cyberattack progression, such as the initial use of social engineering to compromise credentials, followed by malware deployment and the exploitation of vulnerabilities to escalate access. This visual configuration reinforces the notion that threats do not operate in isolation but are strategically chained to maximise their impact and persistence.

**Figure 1:**
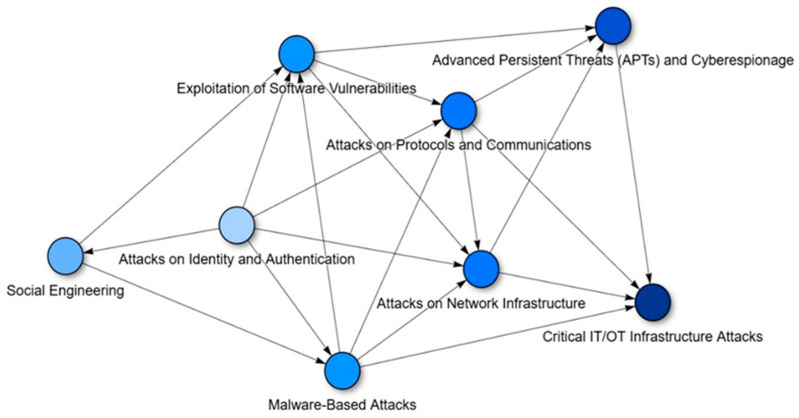
Framework of the proposed taxonomy.

The usefulness of this representation is particularly evident in volatile contexts such as the dark web, where malicious actors combine techniques in a modular and adaptive fashion. In such environments, the framework enables the anticipation of escalation paths and facilitates the identification of critical nodes whose disruption could mitigate the advancement of the attack. Thus, it becomes a valuable tool both for scenario modelling and for prioritising defensive measures in dynamic and decentralised environments. This is illustrated in Figure 1, which presents the proposed taxonomy model and the structure of its inter-group relationships.

## 4. Analysis and Study of Real Cases

The relational model defines 20 directed transitions between attack groups. It shows how one technique enables or reinforces another within the offensive cycle. The relationships are grounded in documented campaigns. In domains such as the dark web, attackers chain techniques to maximise impact evade detection and sustain persistence. Section 4 does not report an experiment. It provides an empirical case-based validation. We map real multistage incidents to the one-step transitions encoded in the matrix. Each transition is supported by at least one documented case. This demonstrates practical applicability in complex settings including critical infrastructures and supply chain contexts.

1. Identity and Authentication Attacks (IAAs) → Social Engineering (SE): The attack known as SIM swapping demonstrates how the compromise of authentication mechanisms can activate social engineering vectors. In this case, attackers manage to duplicate a SIM card by using techniques such as pretexting with the mobile operator, thereby gaining access to SMS messages and calls from the legitimate number. This enables them to bypass verification mechanisms and impersonate the victim or third parties, which facilitates new phases of social manipulation [80].
2. Identity and Authentication Attacks (IAAs) → Malware-Based Attacks (MBAs): Unauthorised access through compromised credentials has been used as an entry point for malware deployment in corporate networks. A representative case is that of the DarkSide ransomware, where attackers gained access to systems using valid credentials, allowing them to deploy malware without triggering alert mechanisms. This pattern demonstrates how authentication attacks can facilitate the execution of malicious code in later phases [81].
3. Identity and Authentication Attacks (IAAs) → Exploiting Software Vulnerabilities (ESVs): The exploitation of software vulnerabilities can be facilitated by the prior compromise of valid identities. In recent incidents, malicious actors used stolen credentials to access enterprise environments and, once inside, exploited unpatched critical vulnerabilities such as those detected in Microsoft Exchange servers. This chaining allowed for persistence and privilege escalation within compromised networks, demonstrating how initial access via compromised identities can serve as a vector for covert software exploitation [82].
4. Identity and Authentication Attacks (IAAs) → Attacks on Protocols and Communications (APCs): Credential compromise can facilitate attacks against communication protocols when attackers use legitimate identities to bypass security controls. A notable case is the 2013 breach suffered by Target, in which a “Pass-the-Hash” attack was executed after accessing the network with credentials stolen from a third-party vendor. This approach enabled lateral movement within the infrastructure without directly exploiting vulnerabilities, manipulating authentication and transmission protocols to maintain persistence and evade detection [83].
5. Identity and Authentication Attacks (IAAs) → Network Infrastructure Attacks (NIA): The use of compromised credentials has enabled botnets such as Mozi to infiltrate exposed network devices, including routers and IP cameras. Once credentials are obtained through brute force or password reuse, attackers compromise the infrastructure to launch distributed denial-of-service (DDoS) attacks or maintain persistent access within the network. This demonstrates how a failure in authentication can trigger direct compromises at the infrastructure layer [84].
6. Social Engineering (SE) → Malware-Based Attacks (MBAs): Social engineering campaigns continue to serve as a key channel for the initial distribution of malware. The Emotet case illustrates this transition; it began as a banking trojan and evolved into a modular malware distribution platform for strains such as TrickBot and QakBot, used by multiple criminal groups. These attacks typically begin with phishing emails impersonating legitimate entities or hijacking compromised email threads. Once the user interacts with the malicious content (attachment or link), malware download is initiated. This pattern highlights how social engineering acts as an entry point for more complex and persistent infections [85].
7. Social Engineering (SE) → Exploiting Software Vulnerabilities (ESVs): In recent malspam campaigns, attackers initiated the chain through social engineering by convincing victims to open RTF documents sent via email. Once opened, these files exploited vulnerabilities in Microsoft Office (such as CVE-2017-11882), enabling the automatic execution of malicious code. This case reflects how an initial manipulation technique can directly lead to the exploitation of previously identified software vulnerabilities [86].
8. Malware-Based Attacks (MBAs) → Attacks on Protocols and Communications (APCs): The global WannaCry outbreak demonstrated how malware can be designed to directly exploit network protocols. After infecting a machine, the worm used the EternalBlue vulnerability to propagate automatically via the SMBv1 protocol, compromising communications between connected systems. This transition shows how a malware-based attack can escalate into protocol-level compromise, amplifying both reach and propagation speed [87].
9. Malware-Based Attacks (MBAs) → Network Infrastructure Attacks (NIA): Malware can serve as a gateway to infrastructure-level compromises, as seen in the Mirai botnet case. This attack used IoT devices infected with malware to launch massive DDoS attacks that directly impacted DNS providers such as Dyn. The large-scale propagation and exploitation of weak configurations directed the attack against key infrastructure, demonstrating how malware-based threats can escalate and disrupt critical network services [88].
10. Malware-Based Attacks (MBAs) → Attacks on Critical IT/OT Infrastructure (CIIA): The NotPetya incident exemplifies how malware can severely impact critical infrastructures. In this case, the malicious code was introduced via a compromised update of the M.E.Doc accounting software and spread using tools like EternalBlue and Mimikatz. The malware directly affected Maersk’s logistics and port operations on a global scale. The infection spread across more than 45,000 devices, causing a total shutdown of operations in 17 port terminals. This demonstrates how malware can evolve into operational sabotage, targeting essential OT infrastructure within global maritime supply chains [89].
11. Malware-Based Attacks (MBAs) → Exploiting Software Vulnerabilities (ESVs): Malware-based attacks are often used as initial vectors to compromise systems and prepare the ground for the exploitation of software vulnerabilities. This is the case with Emotet and TrickBot, which have been used to deliver payloads such as Ryuk, enabling the execution of exploits like EternalBlue. These vulnerabilities allow for the execution of arbitrary code on vulnerable machines without user interaction, thereby facilitating lateral movement and persistence within the compromised network [90].
12. Exploiting Software Vulnerabilities (ESVs) → Attacks on Protocols and Communications (APCs): Breaches originating from vulnerable software (such as the OpenSSL library affected by Heartbleed) enabled attackers to exploit insecure configurations and gain remote access to industrial systems without authentication. This initial software exploitation facilitated subsequent intrusions into critical communication channels (e.g., SCADA and HTTP), clearly illustrating a transition from software exploitation to direct compromise of industrial communication protocols, particularly in internet-exposed ICS environments [91].
13. Exploiting Software Vulnerabilities (ESVs) → Network Infrastructure Attacks (NIAs): The exploitation of vulnerabilities such as Log4Shell (CVE-2021-44228) has shown how a flaw in software libraries can lead to attacks on entire network infrastructures. In this case, remote code execution allowed malicious actors to take control of exposed servers and infiltrate connected systems, enabling lateral movement and manipulation of critical network services. The widespread use of these libraries across both IT and OT environments amplified the attack’s propagation and jeopardised essential components of the digital infrastructure [92].
14. Exploiting Software Vulnerabilities (ESVs) → APTs and Cyberespionage (APT): Sophisticated cyberespionage campaigns have demonstrated the ability to escalate from the initial exploitation of widely used software vulnerabilities (such as those found in SolarWinds Orion) to persistent intrusions in critical infrastructures. These attacks exploit backdoors inserted via legitimate updates, enabling covert and privileged remote access to victim systems. Once inside, threat actors such as APT29 establish hard-to-detect persistence mechanisms, use advanced evasion techniques, and manipulate cloud services to consolidate their presence and extract sensitive information covertly and over extended periods [93].
15. Attacks on Protocols and Communications (APCs) → Network Infrastructure Attacks (NIAs): Attacks targeting communication protocols (such as DNS hijacking) can serve as initial vectors to compromise large-scale network infrastructures. These interferences manipulate legitimate traffic routes to redirect them to malicious servers, facilitating malware deployment or credential theft. In the documented Sea Turtle campaign, DNS records were altered to intercept connections and take control of high-level servers. This initial compromise enabled deeper access to critical infrastructures through persistent redirection techniques [94].
16. Attacks on Protocols and Communications (APCs) → APTs and Cyberespionage (APT): Certain cyberespionage campaigns have begun by manipulating fundamental protocols such as DNS to facilitate covert and persistent access. The Sea Turtle operation is a representative example; attackers modified DNS records of government agencies and technology companies to intercept communications, harvest credentials and then deploy espionage tools to maintain prolonged access to compromised systems. This technique enables the execution of APT activities without directly exploiting software vulnerabilities or conducting overt forced access attempts [95].
17. Attacks on Protocols and Communications (APCs) → Attacks on Critical IT/OT Infrastructure (CIIA): The Industroyer case illustrates how the exploitation of industrial protocols can trigger direct attacks against critical infrastructures. This malware included support for several industrial control protocols (such as IEC 60870-5-101 [96], IEC 60870-5-104 [97], IEC 61850-5 [98] and OPC Data Access [99]), which were used to interact with electrical substation systems, enabling attackers to operate switches and circuit breakers directly. Manipulating these protocols not only facilitated access to systems but also granted operational control over key components of the power grid, disrupting functionality and compromising both physical and logical security [100].
18. Network Infrastructure Attacks (NIAs) → APTs and Cyberespionage (APT): Manipulating network infrastructure can act as a preparatory phase for advanced cyberespionage campaigns. A clear example is VPNFilter, a campaign attributed to APT actors that compromised over 500,000 routers and network devices worldwide. The malware enabled traffic interception, credential theft, and the establishment of persistence for prolonged operations. These capabilities supported covert information gathering and the deployment of targeted attacks against strategic objectives, demonstrating how control over network infrastructure can enable actions characteristic of advanced espionage [101].
19. Network Infrastructure Attacks (NIAs) → Attacks on Critical IT/OT Infrastructure (CIIA): Attacks on network infrastructure are often precursors to deeper compromises of critical infrastructures. A prominent example is the evolution of the BlackEnergy group into GreyEnergy, which (after compromising internet-exposed routers and servers) used this access to deploy backdoors and malware targeting industrial systems. This approach enabled lateral movement into OT networks, affecting strategic sectors such as the energy industry in Ukraine and Poland through tools designed to sabotage operations and conceal their presence [102].
20. APTs and Cyberespionage (APT) → Attacks on Critical IT/OT Infrastructure (CIIA): The Stuxnet case clearly illustrates a transition from a highly sophisticated cyberespionage operation to direct sabotage of critical industrial infrastructures. This malware, attributed to an advanced persistent threat, was specifically designed to infiltrate SCADA systems in Iranian nuclear facilities and silently modify the operational parameters of centrifuges. The intrusion was made possible through zero-day vulnerabilities and stolen digital certificates, enabling privilege escalation and undetected execution of malicious code. Once inside, the worm directly affected physical devices such as Siemens PLCs, altering the behaviour of frequency converters and causing prolonged and untraceable damage, thereby disrupting enriched uranium production. This example shows how an APT campaign can escalate into direct aggression against strategic OT infrastructures without requiring conventional physical attacks [103].

As a consequence of the one-step rule, Identity and Authentication Attacks (IAAs) do not link directly to Attacks on Critical IT/OT Infrastructure (CIIA). Credential compromise may grant initial access or elevate privileges; however, it does not by itself perform the operational action within the critical environment. An intermediate technique is required (for example Network Infrastructure Attacks, Attacks on Protocols and Communications, or Malware-Based Attacks). Accordingly, the model encodes the composed routes IAA → NIA → CIIA, IAA → APC → CIIA, and IAA → MBA → CIIA.

### Illustrative Dark Web Evidence for the Relational Matrix

Dark web ecosystems concentrate operational signals that align with the one-step transitions encoded in our matrix. First, initial access brokerage and credential dumps advertised across marketplaces and forums substantiate IAA → MBA/IAA → NIA; traded valid accounts enable silent malware deployment and facilitate large-scale lateral movement [12,54,60,61,63]. Second, crimeware-as-a-service offerings routinely bundle phishing kits and loaders that deliver modular payloads, supporting SE → MBA and subsequent MBA → APC when malware establishes command-and-control and exfiltration channels [54,60,61,62]. Third, ransomware leak sites and affiliate programmes document chained progressions from access and payload delivery to disruption and extortion, consistent with MBA → CIIA in multistage operations [62,63]. Table 2 summarises the linkage between representative dark web sources and the corresponding initial attacks → facilitated attack.

## 5. Methodology

This study follows a sequential explanatory design to derive and assess a relational matrix of cyberattacks from multistage campaigns with verifiable technical traceability [14,15]. Evidence comes from peer-reviewed literature and technical reports with explicit tactics and operational sequencing. Conceptual alignment relies on established frameworks to preserve terminological consistency and tactical scope [12,23,24].

Extraction relies on a one-step transition rule that decomposes each case into initial attack → facilitated attack mapped to eight attack groups defined in Section 3. This rule reduces semantic ambiguity and prevents multi-hop leaps within a single edge. The outcome is represented as a directed graph and as an 8 × 8 matrix with deduplication per campaign and per transition to preserve cross-case comparability.

Validity is supported through convergence with MITRE ATT&CK and with consolidated models of the offensive cycle and intrusion analysis.

### 5.1. Alignment with MITRE ATT&CK

To ensure terminological consistency and enable independent verification, each of the eight attack groups is mapped to ATT&CK primary tactics and illustrated with representative techniques. Table 3 summarises the operational intent, the dominant ATT&CK tactic(s), and example techniques that typically instantiate each group [12].

### 5.2. Application Method

#### 5.2.1. Purpose and Scope

This subsection defines an operational and reproducible method to apply the relational taxonomy to real incidents or controlled scenarios, in order to identify transitions between attack groups, derive multistage routes, and ground edge acceptance through an explicit, scorable checklist.

#### 5.2.2. Unit of Analysis and One-Step Rule

The unit of analysis is the directed transition between two groups (initial event → facilitated event) encoding an immediate enabling relation. The one-step rule requires each edge to represent a single immediate causal hop. Longer routes are obtained by chaining edges already accepted.

#### 5.2.3. Per-Edge Acceptance Checklist

Each edge is assessed with a six-item binary checklist. Each item scores 1 if satisfied or 0 otherwise. The total score is computed as follows:

Score = d + v + o + a + z + u(1)

- Meaning of each term:
  ○ d (unambiguous direction): Evidence supports the arrow direction (A enables B) and rules out reverse causality or mere co-occurrence.
  ○ v (technical feasibility): The transition is technically plausible given attacker capabilities, environment, and existing controls.
  ○ o (observability): Data sources exist that can log the hop or its traces, even if not all are present in the case.
  ○ a (ATT&CK mapping at origin): The initial event aligns with concrete ATT&CK tactics and techniques (verifiable IDs).
  ○ z (ATT&CK mapping at destination): The facilitated event aligns with concrete ATT&CK tactics and techniques (verifiable IDs).
  ○ u (operational usefulness): The transition provides practical value for detection, containment, or control prioritisation in an SOC.
- Decision rule:
  ○ Acceptance threshold: accept the edge if score ≥ 5.
  ○ Labels:
    ▪ 6/6: when all six items are satisfied.
    ▪ 5/6: when exactly one item fails.
    ▪ <5/6: edge not accepted (documented as hypothesis or insufficient evidence).
  ○ The ≥5 threshold balances parsimony (avoids weak hops) and operational robustness (tolerates one missing item under incomplete telemetry). The 6/6 label identifies canonical edges (strong evidence and consensus) that serve as references and cores of frequent routes. The 5/6 label captures strong edges typical of real-world settings where one of the six conditions may be uncertain or partially observable.

#### 5.2.4. Inputs

1. Chronological incident narrative and artefacts.
2. Technical evidence (logs, alerts, forensics, IOCs).
3. Group-level ATT&CK alignment table.
4. Templates: 8 × 8 matrix, per-edge checklist, edge → ATT&CK table.

#### 5.2.5. Seven-Step Protocol

1. Define scope: time window, assets, evidence sources, inclusion criteria.
2. Label by group: assign each event to the appropriate group with ATT&CK support.
3. Extract transitions: identify initial attack→ facilitated attack linked by technical causality and temporal order.
4. Assess with checklist: score the six items, compute score, decide acceptance.
5. Populate the 8 × 8 matrix: mark 1 in (origin, destination) for accepted edges; de-duplicate per campaign.
6. Graph and routes: build the directed graph with accepted edges; derive routes by chaining, respecting the one-step rule.
7. Edge → ATT&CK and report: document tactic(s) and technique(s) at origin and destination, detection exemplars, and controls.

#### 5.2.6. Quality Criteria and Bias Control

- Inter-rater consistency: double coding of a sample and consensus resolution.
- Traceable reasoning: each checklist tick links to verifiable evidence.
- No causal conflation: distinguish co-occurrence from enablement and justify arrow direction.
- Parsimony: prefer immediate transitions; longer paths are modelled by chaining.

#### 5.2.7. Minimal Deliverables

- Updated 8 × 8 matrix.
- Edge table with checklist and totals.
- Directed graph with derived routes.
- Edge → ATT&CK table to support detection and hunting.
- Executive summary with cut-points and control prioritisation.

## 6. Taxonomy Validation

The taxonomy is validated through two complementary strategies. First, a worked example executed by the authors applies the method step by step to verify internal coherence, traceability, and transition consistency, and to explain why and how it should be used in a representative scenario. Second, an independent panel of five experts in cyber threat intelligence, security engineering, and SOC operations evaluates the acceptance criteria, the stability of the one-step rule, and the operational clarity of the matrix. Together, these strands provide construct and content validity and demonstrate the reproducibility of the procedure. Together these strands provide construct validity and content validity and demonstrate reproducibility of the procedure. Inter-rater agreement is estimated using a kappa coefficient and disagreements and their resolution by consensus are recorded.

### 6.1. Worked Example

- Context. A spear phishing campaign delivers a payload with persistence and C2, progresses towards network infrastructure, and culminates in availability impact.
- Timeline:
  ○ Day 1: Targeted email with malicious attachment.
  ○ Day 2: User execution and persistence.
  ○ Day 3: Outbound C2 and initial exfiltration.
  ○ Day 4: DNS manipulation and progression to critical systems.
  ○ Day 5: Impact on availability.
- Evaluated transitions, formula application and decision:
  1. SE → MBA
    ○ Formula application (1)
      ▪ d = 1 (clear temporal and causal chain from email to execution)
      ▪ v = 1 (documented spear phishing and user execution techniques)
      ▪ o = 1 (email gateway, EDR, autostart events)
      ▪ a = 1 (TA0001/T1566; T1204)
      ▪ z = 1 (TA0002/TA0003; T1059, T1547)
      ▪ u = 1 (strong control leverage at mail and execution layers)
      ▪ score = 6 → 6/6 → Accepted
    ○ Operational notes
      ▪ Advanced filtering and execution hardening reduce this hop.
  2. MBA → APC
    ○ Formula application (1)
      ▪ d = 1 (payload establishes C2 and initiates exfiltration)
      ▪ v = 1 (beaconing and encrypted channels are feasible)
      ▪ o = 1 (proxy, NDR, exfiltration patterns)
      ▪ a = 1 (TA0002/TA0003; T1059, T1053)
      ▪ z = 1 (TA0011/TA0010; T1071, T1573, T1041)
      ▪ u = 0 (immature controls to fully suppress the channel in this setting)
      ▪ score = 5 → 5/6 → Accepted
    ○ Operational notes
      ▪ Prioritise C2 detection and egress limitations.
  3. APC → NIA
    ○ Formula application (1)
      ▪ d = 1 (network-level control enables progression to infrastructure assets)
      ▪ v = 1 (DNS/remote services and tool transfer are viable)
      ▪ o = 0 (partial telemetry in network devices)
      ▪ a = 1 (TA0011; T1071.004)
      ▪ z = 1 (TA0008; T1021, T1570)
      ▪ u = 1 (segmentation and DNS control as cut-points)
      ▪ score = 5 → 5/6 → Accepted
    ○ Operational notes
      ▪ Improve visibility across infrastructure devices.
  4. NIA → CIIA
    ○ Formula application (1)
      ▪ d = 1 (network changes precipitate impact)
      ▪ v = 1 (plausible impact techniques against services)
      ▪ o = 1 (downtime logs and config changes)
      ▪ a = 1 (TA0008; T1021/T1040)
      ▪ z = 0 (no specific ATT&CK impact sub-technique cited and insufficient operational evidence)
      ▪ u = 1 (BCP and containment playbooks)
      ▪ score = 5 → 5/6 → Accepted
    ○ Operational notes
      ▪ Rehearse recovery and block impact actions.
  5. MBA → CIIA (direct variant)
    ○ Formula application (1)
      ▪ d = 1 (payload embeds direct impact capability)
      ▪ v = 1 (e.g., encryption for impact)
      ▪ o = 1 (EDR/AM and system events)
      ▪ a = 1 (TA0002/TA0003; T1059/T1543)
      ▪ z = 0 (generic impact description without a precise sub-technique or supporting telemetry)
      ▪ u = 1 (specific controls against execution/impact)
      ▪ score = 5 → 5/6 → Accepted
    ○ Operational notes
      ▪ Harden execution policies and protect immutable backups.
- Derived routes:
  (a) Route A (primary): SE → MBA → APC → NIA → CIIA
    ○ Priority cut-points:
      ▪ SE → MBA (mail and execution policies)
      ▪ MBA → APC (C2/exfiltration detection)
      ▪ APC → NIA (segmentation and DNS)
  (b) Route B (variant): SE → MBA → CIIA
    ○ Priority cut-points:
      ▪ SE→MBA (mail)
      ▪ MBA→CIIA (execution blocking and recovery protection)
- Edge → ATT&CK table
  ○ The following Table 4 summarises the validated transitions from the worked example. Each row presents the ATT&CK tactic at origin and at destination together with representative techniques that characterise the operational hop. Its purpose is to ease route interpretation guide detection and threat hunting and prioritise controls without claiming exhaustiveness.

### 6.2. Expert Validation

To empirically assess whether the proposed method—operationalised through the taxonomy and the 8 × 8 matrix—provides sufficient coverage and practical utility, a five-point Likert survey (1 = not adequate; 5 = highly adequate) was administered to five independent experts (CTI, security engineering and SOC operations). The instrument targets, within the coding and per-edge decision workflow, both the methodological coverage of the artefacts (checklists, rules, and criteria) and their perceived operational usefulness. Analysis is restricted to descriptive statistics (mean and standard deviation) and an item-level decision (Keep/Revise) oriented to practical validation of the method.

#### 6.2.1. Acceptance Criteria and Methodological Justification

- Metric selection (means and SD for Likert items): In applied evaluation, reporting means and standard deviations for Likert-type items is appropriate for transparent, decision-oriented summarisation [104,105].
- Robustness of parametric summaries: Parametric summaries of Likert-type data are empirically robust to modest violations of intervality and normality, legitimising their use for descriptive synthesis and design decisions [106].
- Interval-based interpretation on five-point scales: A five-point scale has an approximate category width of 0.80, which enables meaningful interpretation of means in bands and motivates an SD ≤ 0.8 criterion as evidence of concentrated responses within a single band [107].
- Operational decision rule:
  ○ Keep if mean ≥ 4.0 and SD ≤ 0.8 (high/very high agreement with limited dispersion).
  ○ Minor revise if 3.5 ≤ mean < 4.0 and SD ≤ 0.8 (adequate agreement; small clarifications recommended).
  ○ Revise if mean < 3.5 or SD > 0.8 (insufficient agreement and/or diffuse responses).

#### 6.2.2. Items Evaluated by the Expert Panel (Q1–Q8)

The panel assessed the method against eight operational properties that collectively reflect its validity in use.

○ Q1—Process flow (clarity): comprehensibility and executability of the methodological sequence.
○ Q2—Checklist coverage: adequacy of the six items (d, v, o, a, z, u) for per-edge decisions.
○ Q3—Granularity and evidence: sufficiency of operational detail and ease of attaching verifiable evidence.
○ Q4—Preconditions and criteria: clarity and applicability of preconditions and progress/rework gates.
○ Q5—Rule and threshold (≥5/6): practical utility of the threshold and its labelling for prioritisation and traceability.
○ Q6—ATT&CK mappings: coherence and operational usefulness of origin/destination mappings.
○ Q7—Traceability and perceived reproducibility: clarity of the item–evidence–decision trail and expected rater convergence.
○ Q8—Internal coherence and applicability: fit across components and platform-independent execution.

#### 6.2.3. Expert Responses

Table 5 presents the raw Likert responses (1–5) of the five experts to each of the eight items.

#### 6.2.4. Item Results and Decision for the Method

Table 6 reports the aggregated results (mean, SD) for each item and the corresponding decision under the pre-specified rule.

#### 6.2.5. Validation Conclusion

All items meet mean ≥ 4.0 with SD ≤ 0.8, satisfying the pre-specified operational thresholds. Taken together—with accepted practice for reporting means/SD on Likert items, the robustness of parametric summaries for Likert-type data, and interval-based interpretation on five-point scales—the findings support retaining the current design. The method demonstrates sufficient coverage and operational usefulness for auditable, consistent per-edge decisions.

## 7. Discussion

In this study, the proposed method operationalises the relational taxonomy through a verifiable workflow: It identifies immediate transitions (initial → facilitated) [108], via a one-step rule, applies acceptance criteria to decide each edge, and projects accepted edges onto a directed graph and an 8 × 8 matrix with per-campaign deduplication. This sequence renders the coding auditable, prevents conflating co-occurrence with enablement, and preserves terminological consistency by anchoring every hop to ATT&CK to verify source and destination.

The results show that the matrix synthesises 20 direct relations across the eight groups and enables the reconstruction of tactical progressions observed in multistage campaigns. In the worked example, the routes SE → MBA → APC → NIA → CIIA and SE → MBA → CIIA are accepted with 5/6–6/6, identifying prioritisation points (mail and execution, C2/exfiltration, segmentation/DNS) from evidence (email gateway, EDR, NDR) and per-edge ATT&CK mappings. This pattern is consistent with contemporary descriptions of escalations in which phishing delivers payloads, C2 is established, and lateral movement is enabled until availability impact materialises [28].

Empirical corroboration with documented incidents reinforces the external validity of these transitions. NotPetya exemplifies MBA → CIIA through escalation to operational impact in logistics and port terminals after propagation via tools such as EternalBlue and Mimikatz [89]. Sea Turtle shows APC → NIA and APC → APT by manipulating DNS (record hijacking and redirections) prior to consolidating persistent access and espionage [94,95]. Industroyer evidences APC → CIIA by operating directly on industrial protocols (IEC 60870-5-101 [96], IEC 60870-5-104 [97], IEC 61850-5 [98] and OPC Data Access [99]) and actuating substation equipment [100]. VPNFilter supports NIA → APT by compromising hundreds of thousands of routers, enabling interception and persistence for espionage campaigns [101]. GreyEnergy illustrates NIA → CIIA by pivoting from exposed infrastructure into OT networks in the energy sector [102]. In SolarWinds, the ESV → APT transition materialises through a backdoor in legitimate updates and sustained covert persistence [93]. At exploitation level, Heartbleed and Log4Shell substantiate ESV → APC and ESV → NIA, respectively, enabling remote access, pivoting, and control of network services [91,92].

Findings from the dark web ecosystem help explain the frequency of initial routes. Access brokerage and credential dumps support IAA → MBA/IAA → NIA by enabling silent malware deployment and lateral movement; crimeware kits and loaders with C2 sustain SE → MBA/MBA → APC by chaining payload delivery with channel establishment and exfiltration; leak sites and affiliate programmes document complete chains compatible with MBA → CIIA [12,54,58,60,61,63]. Section Illustrative Dark Web Evidence for the Relational Matrix and its correspondence table link these observations to specific transitions and their operational justification, aligning open-source evidence with the resulting matrix.

Expert validation supports the method’s methodological coverage and operational utility: item means (≥4.0) with SD ≤ 0.71 meet the pre-specified criteria and lead to a Keep decision across process, evidence, preconditions, rule, ATT&CK mappings, traceability, and internal coherence [15,104,105,106,107]. This support converges with the metric justification for Likert scales and the need for actionable decisions in edge coding, reinforcing the use of 6/6 evaluations as cores of frequent routes and 5/6 as a robust standard under incomplete telemetry.

In conclusion, the proposed method demonstrates explanatory and operational capability to derive routes from verifiable immediate transitions, with ATT&CK anchoring and evidence from real incidents, and with expert backing that supports consistent application to campaign analysis and control prioritisation.

## 8. Conclusions

This study presents a relational taxonomy of cyberattacks grounded in the identification of operational interdependencies across threat vectors. Unlike traditional hierarchical schemes, the model offers a closer representation of the sequential and modular nature of attack techniques in complex scenarios.

From a social perspective, the relational structure is particularly valuable in distributed and collaborative ecosystems such as the dark web. Understanding how techniques are linked enhances public awareness and supports the design of training programmes aligned with real risk, improving early detection of fraud patterns and behaviour-manipulation campaigns.

At the organisational level, the proposal provides security teams with a tool to strengthen defensive posture by enabling resource prioritisation based on plausible attack paths, anticipates tactical escalations, and improves incident lifecycle management. It also fosters alignment of response capabilities with more advanced and adaptive threat intelligence practices.

In the technological domain, the methodology adds a structured layer that models one-step transitions across phases and techniques and promotes interoperability with intelligence-sharing platforms. This layer produces traceable, consumable outputs that support the automation of correlations, the identification of recurrent sequences, and faster detection and mitigation, not as a replacement for existing systems, but as a complementary decision framework.

Validation of the taxonomy confirms the methodological robustness of the model and the clarity of the method. The results show internal consistency and stability of the one-step rule in heterogeneous scenarios. The matrix preserves traceability among techniques and maintains scope under contextual variation. The method exhibits sufficient reproducibility and adequate documentation for unambiguous application by third parties. Integration with MITRE ATT&CK ensures stable mapping and a well-bounded domain, and the design supports incremental updates without loss of traceability.

The current scope is limited to immediate single-step transitions. It does not yet incorporate quantitative attributes, precise temporal windows, or contextual conditions such as asset type or required privilege. These limitations operate as design boundaries and guide future extensions.

Accordingly, three lines of future research are proposed. The first line is to formalise the taxonomy as an ontology that incorporates quantitative attributes, precise temporal windows, and contextual conditions such as asset type and required privilege, enabling serialisation in exchange languages and integration with collaborative platforms. The second line is to extend the approach towards near real-time dynamic analysis over operational telemetry, supported by open-source intelligence and signals from clandestine forums and markets, in order to enrich the interpretation of transitions and strengthen decision-making. Finally, the third line is to link the modular architecture with dynamic clustering techniques and AI-assisted detection, leveraging consumable, traceable artefacts to reinforce traceability and the effectiveness of analytic pipelines and to promote reproducibility.

## Figures and Tables

**Table 1:** Relational matrix of direct cyberattack dependencies.

Initial Attack ↓/Facilitated Attack →	SE	MBA	ESV	APC	NIA	CIIA	APT
IAA	1	1	1	1	1	0	0
SE	0	1	1	0	0	0	0
MBA	0	0	1	1	1	1	0
ESV	0	0	0	1	1	0	1
APC	0	0	0	0	1	1	1
NIA	0	0	0	0	0	1	1
APT	0	0	0	0	0	1	0

**Table 2:** Dark web sources mapped to one-step transitions.

Evidence Source	Observed Phenomenon	Mapped Transition(s)	Justification
Marketplace datasets and forum studies [9,54,60,61]	Sale of valid accounts and initial access; credential dumps.	IAA → MBA IAA → NIA	Traded credentials enable stealthy payload deployment and lateral movement.
Crimeware-as-a-service/kit ecosystems [54,60,61,62]	Phishing kits and loaders delivering modular payloads with C2.	SE → MBA MBA → APC	Social delivery triggers execution; malware establishes C2 and exfiltration.
Ransomware leak sites and affiliate operations [62,63]	End-to-end campaigns from access to extortion.	MBA → CIIA	Payloads impair operations; leak sites document sequencing and impact.
Tor domain centrality/influence [58]	Concentration of brokers, kits, and monetisation hubs.	IAA → MBA IAA → NIA SE → MBA MBA → APC MBA → CIIA	Structural centrality explains frequent co-occurrence of enabling steps.

**Table 3:** Alignment of the eight groups with MITRE ATT&CK.

Taxonomy Group	Primary ATT&CK Tactic(s)	ATT&CK Techniques
SE	TA0001 Initial Access	T1189: Drive-by Compromise T1204.001: User Execution Malicious Link T1204.002: User Execution Malicious File T1566: Phishing T1566.001: Spear phishing Attachment T1566.002: Spear phishing Link T1566.003: Spear phishing via Service
IAA	TA0004: Privilege Escalation TA0006: Credential Access	T1003: Credential Dumping T1003.001: OS Credential Dumping LSASS Memory T1078: Valid Accounts T1110: Brute Force T1110.003: Password Spraying T1550.002: Pass-the-Hash T1550.003: Pass-the-Ticket T1621: Multifactor Authentication Interception
MBA	TA0002 Execution TA0003 Persistence	T1053: Scheduled Task Job T1059: Command and Scripting Interpreter T1059.001: PowerShell T1059.006: Python T1543.003: Windows Service T1547.001: Registry Run Keys Startup Folder T1574.001: DLL Search Order Hijacking T1129: Shared Modules
NIA	TA0007 Discovery TA0008 Lateral Movement	T1018: Remote System Discovery T1021: Remote Services T1021.001: RDP T1021.002: SMB Windows Admin Shares T1040: Network Sniffing T1570: Lateral Tool Transfer T1557.002: ARP Cache Poisoning
ESV	TA0001 Initial Access TA0004 Privilege Escalation	T1068: Exploitation for Privilege Escalation T1189: Drive-by Compromise T1190: Exploit Public-Facing Application T1203: Exploitation for Client Execution T1210: Exploit Remote Services
APC	TA0010 Exfiltration TA0011 Command and Control	T1041: Exfiltration Over C2 Channel T1048: Exfiltration Over Unencrypted Obfuscated Non-C2 Protocol T1071: Application Layer Protocol T1071.001: Web Protocols T1071.004: DNS T1567: Exfiltration Over Web Services T1573: Encrypted Channel
APT	TA0003 Persistence TA0005 Defence Evasion	T1027: Obfuscated Compressed Files and Information T1078: Valid Accounts T1218: Signed Binary Proxy Execution T1547: Boot or Logon Autostart Execution T1562.001: Disable Security Tools T1564: Hide Artefacts
CIIA	TA0040 Impact	T1485: Data Destruction T1486: Data Encrypted for Impact T1489: Service Stop T1490: Inhibit System Recovery T1496: Resource Hijacking T1499: Endpoint Denial of Service

**Table 4:** Edge → ATT&CK detection mapping table.

Edge	Tactic at Origin	Example Technique(s)	Tactic at Destination	Example Technique(s)
SE → MBA	TA0001 Initial Access	T1204 User Execution T1566.001 Spear phishing Attachment	TA0002 Execution TA0003 Persistence	T1059 Command and Scripting Interpreter T1547 Boot or Logon Autostart Execution
MBA → APC	TA0002 Execution TA0003 Persistence	T1053 Scheduled Task/Job T1059 Command and Scripting Interpreter	TA0010 Exfiltration TA0011 Command and Control	T1041 Exfiltration Over C2 Channel T1071 Application Layer Protocol T1573 Encrypted Channel
APC → NIA	TA0011 Command and Control	T1071.004 Application Layer Protocol: DNS	TA0008 Lateral Movement	T1021 Remote Services T1570 Lateral Tool Transfer
NIA → CIIA	TA0008 Lateral Movement	T1021 Remote Services T1563 Remote Service Session Hijacking	TA0040 Impact	T1486 Data Encrypted for Impact T1490 Inhibit System Recovery
MBA → CIIA	TA0002 Execution TA0003 Persistence	T1059 Command and Scripting Interpreter T1543 Create or Modify System Process	TA0040 Impact	T1485 Data Destruction T1486 Data Encrypted for Impact T1499 Endpoint Denial of Service

**Table 5:** Expert responses.

Expert	Q1	Q2	Q3	Q4	Q5	Q6	Q7	Q8
E01	5	5	4	4	5	5	5	4
E02	4	4	3	4	4	3	4	4
E03	5	4	5	5	5	4	4	4
E04	4	4	4	4	4	4	5	4
E05	5	4	4	5	5	4	4	5

**Table 6:** Item results and decision for the method.

Code	Short Label	Mean	SD	Decision
Q1	Process flow (clarity)	4.60	0.55	Keep
Q2	Checklist coverage	4.20	0.45	Keep
Q3	Granularity and evidence	4.00	0.71	Keep
Q4	Preconditions and criteria	4.40	0.55	Keep
Q5	Rule and threshold (≥5/6)	4.60	0.55	Keep
Q6	ATT&CK mappings	4.00	0.71	Keep
Q7	Traceability and perceived reproducibility	4.40	0.55	Keep
Q8	Internal coherence and applicability	4.20	0.45	Keep

## Data Availability

The original contributions presented in this study are included in the article. Further inquiries can be directed to the corresponding author.
